# Effects of combining manual therapy, neck muscle exercises, and therapeutic pain neuroscience education in patients with migraine: a study protocol for a randomized clinical trial

**DOI:** 10.1186/s12883-021-02290-w

**Published:** 2021-06-29

**Authors:** Gabriella de Almeida Tolentino, Lidiane Lima Florencio, Carina Ferreira Pinheiro, Fabíola Dach, César Fernández-de-las-Peñas, Débora Bevilaqua-Grossi

**Affiliations:** 1 Department of Health Sciences – Ribeirão Preto Medical School, University of São Paulo, 3900, Bandeirantes Avenue – Monte Alegre, Ribeirão Preto, SP 14049-900 Brazil; 2 Department of Physical Therapy, Occupational Therapy, Rehabilitation and Physical Medicine, Universidad Rey Juan Carlos, Alcorcón, Spain; 3 Department of Neurosciences and Behavioral Sciences – Ribeirão Preto Medical School, University of São Paulo, Ribeirão Preto, SP Brazil

**Keywords:** Headache, Neck pain, Combined modality therapy, Physical therapy modalities

## Abstract

**Background:**

Non-pharmacological approaches for the management of migraine exhibit low to moderate effectiveness due to a lack of high-quality randomized clinical trials. In fact, previous studies applied isolated techniques, which were not representative of common clinical practice. A multimodal approach for migraine may benefit these patients more than isolated approaches. This randomized clinical trial aims to determine the effectiveness of a multimodal protocol combining manual therapy, exercise, and therapeutic pain neuroscience education versus the application of manual therapy or pain neuroscience education alone in patients with migraine.

**Methods:**

This clinical trial will include 75 individuals of both sexes, aged between 18 and 55 years, with migraine. Participants will be randomized into three groups: the therapeutic pain neuroscience education (TPNE; *n* = 25) group, the manual therapy (MT; *n* = 25) group, and the multimodal (MM; *n* = 25) group. The TPNE group will receive one orientation session on migraine and pain self-management, and recommendations for daily active stretching and walking, with subsequent therapist monitoring. The MT group will receive manual therapies targeting musculoskeletal disorders of the cervical spine. The MM group will receive manual therapies targeting musculoskeletal disorders of the cervical spine, active neck exercises, and therapeutic pain neuroscience education. The treatment period will last 12 weeks. The primary outcome will be the headache impact, measured using the Headache Impact Test (HIT-6). Secondary outcomes will include migraine frequency and intensity, cervical mobility and strength parameters, neck pain-related disability, kinesiophobia, cutaneous allodynia, pain-related catastrophizing, quality of life, and self-perception of change. All outcomes will be evaluated at the fourth, eighth, and twelfth weeks of the treatment period. Primary and secondary clinical outcomes, such as headache impact, frequency, and intensity, will also be evaluated at the 1-, 2-, and 4-month follow-ups.

**Discussion:**

The results of this randomized clinical trial may provide high-quality clinical evidence of the effects of non-pharmacological treatment options for the management of migraine.

**Trial registration:**

This study was registered under the access code RBR-7s22c75 in the *Registro Brasileiro de Ensaios Clínicos* (ReBEC) in December 2020.

## Background

Migraine, a neurological disease, is considered the second leading cause of disability, affecting one billion people worldwide [1]. This disease is characterized by attacks of unilateral, recurrent, pulsating headache of moderate to severe intensity that lasts for 4–72 h and increases with daily routine or physical activity, and may be accompanied by nausea and/or photophobia and phonophobia [2]. Currently, pharmacological treatment has the highest level of evidence for managing migraine [3]. However, non-pharmacological approaches [4, 5], for e.g., acupuncture, self-management techniques, pain neuroscience education, relaxation strategies, mindfulness, and physical therapy [5–7], are also extensively used by patients.

Physiotherapy, a conservative, non-invasive, non-pharmacological intervention, is used as an adjuvant to pharmacological treatment of headache, and presents evidence of decreasing the frequency and intensity of migraine attacks [8–11]. Manual therapy with trigger point release, spinal mobilization/manipulation, and cervical stretching, present similar evidence to pharmacological therapy for reducing the frequency and intensity of headache [9, 10, 12, 13]. Moreover, there is also moderate- to low-level evidence for electrotherapy [14] and aerobic exercises [11, 15] for migraine. However, these techniques have been tested only separately, without associating them with different headache management approaches.

Therapeutic pain neuroscience education has also been shown to be an evidence-based clinical approach that provides additional benefits, when associated with pharmacological therapy, for reducing headache frequency [16–18]. Kindelan-Calvo et al. [19] found strong evidence supporting an improvement in disability and a decrease in the frequency of headaches after the application of therapeutic pain neuroscience education in migraineurs.

Craniocervical exercises may also be beneficial for patients with migraine, as they have proven to be effective in individuals with neck pain and other headaches associated with cervical spine impairments [20–22]. However, no systematic review has determined its effectiveness in this population. Considering the decrease in cervical muscular performance [23, 24] and cervical muscle resistance [25] in women with migraine, these patients could also benefit from associated exercise programs. It has already been proven that different musculoskeletal and functional dysfunctions accompany migraine headaches. Furthermore, it has been suggested that patients with migraine could benefit from multimodal treatment that includes relaxation techniques, manual therapy, stretching, and active neck exercises, in addition to therapeutic pain neuroscience education. No study has investigated the additional effects of these interventions in a population with migraine.

The primary aim of this randomized clinical trial will be to determine the effectiveness of a multimodal approach combining manual therapy, exercises, and therapeutic neuroscience pain education on the severity of headache in individuals with migraine. The secondary aims will be to determine its effectiveness on headache frequency (days/month), intensity, pressure pain sensitivity, cervical mobility, and related functions. We will also analyze if there were effects on neck pain-related disability, cutaneous allodynia, kinesiophobia, pain-related catastrophizing, global self-perception of change, and the patient’s quality of life. Thus, we hypothesize that the multimodal physical therapy protocol, which associates multiple interventions with patient education, will help reduce migraine disability, when compared to the application of each intervention alone.

## Methods

### Design

A three-armed, parallel-group, randomized clinical trial with a 12-week treatment protocol and follow-up assessments after 1, 2, and 4 months will be conducted according to CONSORT guidelines [26]. This treatment protocol will be described in accordance with the SPIRIT recommendations [27].

### Setting

The study will adhere to the guidelines provided by the International Headache Society (IHS) for clinical trials, with respect to the inclusion and exclusion criteria, outcome measures, and statistical analysis. The study protocol has been approved by the local ethics committee (CAAE 36575220.5.0000.5440), and the trial was registered under the access code RBR-7s22c75 in the *Registro Brasileiro de Ensaios Clínicos* (ReBEC) in December 2020. All participants will receive and sign an informed consent form before the beginning of the trial.

The evaluations and interventions will be conducted at the *Hospital das Clínicas* of the Ribeirão Preto Medical School of the University of São Paulo (HCFMRP-USP) and the Laboratory of Evaluation of Posture and Human Movement of the Ribeirão Preto Medical School. The study will include three primary researchers; two will be responsible for data collection, and the third one will be responsible for treatments.

### Participants

Inclusion criteria are individuals of both sexes, aged between 18 and 55 years, and diagnosed with migraine according to the 3rd edition of the IHS (ICHD-III), with at least three days of migraine per month in the previous month.

Exclusion criteria will include other concomitant primary/secondary headaches, history of facial or neck trauma, history of cervical disc herniation or cervical osteoarthritis, any systemic degenerative disease (e.g., rheumatoid arthritis, lupus erythematosus), pregnancy, physiotherapeutic treatment of the craniocervical region in the previous year, and/or having started a new pharmacological treatment for migraine in the 3 months prior to participating in the trial.

### Interventions

#### Therapeutic pain neuroscience education (TPNE)

Twenty-five participants will be randomly assigned to this group, which will be the active control group. They will attend a single appointment of approximately 60 min aimed at addressing pain neuroscience education, adapted from the protocol by Holroyd et al. [16] and complemented with materials from the Pain Research group (Brazil) [28] for neuroscience-based pain education. The therapist, with 4 years of clinical experience in this approach, will instruct the subjects on the pathophysiology of migraine, identification of prodromal warning signs and possible triggers, management of disease-related behavioral skills, and training relaxation techniques (stretching, deep breathing, progressive muscle relaxation, and relaxation images) in a single session, of approximately 50 min [16]. In addition, the participants will receive educational material containing home-based recommendations for daily activities during the 12 weeks of treatment, including those on self-management of migraine attacks, self-massage, active stretching, and the practice of physical activities, such as walking for 30–40 min. Patients reporting vestibular symptoms and/or a history of falls will also be instructed to perform basic home exercises to control balance, with both eye and head movements [29]. After this session, participants will be contacted weekly by the therapist through phone calls or mobile messages to assess their orientations adherence and solve any questions or problems regarding the exercises.

#### Manual therapy (MT)

Twenty-five participants will be randomly assigned to this group and will receive treatment once a week, for 12 weeks, with each session lasting approximately 40 min, from a physical therapist with 4 years of clinical experience. The protocol of this group was based on the protocol by Bevilaqua-Grossi et al. [9], with no home-based orientations.

It consist of the following five steps: 1, Diaphragmatic respiratory training for 5 min guided by the therapist [9, 30], combining a profound inhalation using the diaphragm and a gentle exhalation which ends up with a conscious contraction of abdominal muscles; 2, cervical spine traction mobilization for another 5 min, with slow, progressive, and intermittent traction of the occiput timed with the diaphragmatic breathing [31]; 3, deep massage and myofascial release of craniocervical muscles for 15 min, especially the suboccipital, upper trapezius, sternocleidomastoid, and scalene muscles; 4, 10 min to trigger points ischemic compression, applying a 90 s compression at a maximum of six trigger points identified in the craniocervical muscles according to the most recent criteria [32]. The points to be treated will be chosen according to the intensity of pain at that point (0–10) and reproduction of headache symptoms. The session will end up at the fifth step, which consist of three repetitions in each direction of passive stretching of neck muscles (flexion, lateral flexion, and rotation associated with ipsilateral flexion) sustained by 30 s.

#### Multimodal group

The participants randomly allocated to the multimodal group will receive treatment from a physical therapist with 4 years of clinical experience, once a week, for 12 weeks, with each session lasting approximately 50 min. The proposed protocol is based on the available evidence using isolated treatments for the management of the most prevalent musculoskeletal disorders observed in patients with migraine [9, 33]. Treatment will consist of combined techniques for relaxation, stretching, and myofascial release, focusing primarily on craniocervical muscles, with an exercise program for the cervical muscles as proposed by Falla et al. [33], as well as therapeutic pain neuroscience education. Participants will be instructed to practice cervical exercises once a day during the treatment period, with weekly reminders sent via SMS, WhatsApp®, or e-mail to perform these exercises.

The first treatment session will cover the same therapeutic pain neuroscience education program as the TPNE group [16, 28]. During treatment, time will be reserved during the session to reinforce and continue the pain education. The participants will receive in the initial session an educational material containing home-based recommendations for daily activities during the 12 weeks of treatment.

The participants will receive educational material containing home-based recommendations for daily activities during the 12 weeks of treatment, including those on self-management of migraine attacks, self-massage, active stretching, and the practice of physical activities, such as walking for 30–40 min.

From the second session, the protocol will follow the same interventions mentioned in the MT, changing only the time of massage/myofascial release to five min, adding 15 min to the active flexor and extensor muscle exercises [33], and 5 final minute of general orientation [16, 19]. The exercise program will consist of two stages, adapted from the protocol described by Falla et al. [33]. The first stage will last 8 weeks, consisting of flexion and extension exercises of the cervical spine. The incremental craniocervical flexion will be performed in a relaxed supine position, without a pillow, in two sets of 10 repetitions with 10 s of support. For craniocervical extension, participants will perform craniocervical extension, flexion, and rotation in a supine position with elbows supported, keeping the cervical spine neutral. Two sets of 10 repetitions will be carried out, which can progress to up to three sets of 10 repetitions. The series, repetitions, or time of support in the posture will increase according to the patient’s tolerance. There will be an adjustment of the schedule if the participant is unable to perform it. The second stage will be 4 weeks in duration and will feature high-load exercises, both for flexion and extension, with the head weight as the load. Head flexion will be preceded by cervical flexion in the supine position, in three series of the 15 repetitions, and maintained until the end of the protocol. For the neck extension exercise, the patients will be instructed to keep the craniocervical region in the middle position while extending the cervical region, which will be performed in 4-pt kneeling. This will also be done in three series of the 15 repetitions until completion of the protocol.

The general orientation time will be dedicated to complementing the patient’s therapeutic pain neuroscience education, reinforcing the orientation and management approach of the first session, and to solve the possible existing doubts of the participants.

Individuals from all intervention’s groups will be advised not to start any new treatment and not change their current medication use during the trial. There will be no possibility to change allocated interventions and treatments will be discontinued only if the participant request.

### Outcomes

#### Primary

The primary outcome will be headache impact, measured using the Headache Impact Test (HIT-6), requiring a decrease of at least 6 points in the headache-related impact measured by the HIT-6 [34], which will be applied at the baseline, and at four, eight, and 12 weeks of treatment, and at follow-ups at 1, 2, and 4 months.

#### Secondary

The secondary outcomes will be as follows: frequency of migraine, intensity of migraine, pressure pain threshold, cervical range of motion, cervical muscle strength, upper cervical mobility, cervical muscle performance, disability related to neck pain, cutaneous allodynia, kinesiophobia, pain-related catastrophizing, quality of life, and perception of clinical change. All secondary outcomes will be measured at the fourth, eighth, and twelfth week. The frequency and intensity of migraine will also be evaluated at the 1-, 2-, and 4-month follow-ups.

### Sample size

The sample size was calculated for the primary outcome, the HIT-6. It was based on a mean reduction of six points and a standard deviation of 6.03, while adopting a significance level (α) of 0.05 and power of 90%. We estimated 21 individuals per group, and considering a 15% loss to follow-up, 25 individuals per group will be required. Therefore, 75 individuals will be included in this clinical trial. An effect size of ≥ 6 points at HIT-6 is the minimum clinically important difference (MCID) for this scale [34]. The standard deviation was derived from a sample of nine volunteers from our database of previous studies.

### Recruitment

Participants will be recruited from the Headache Outpatient Clinic of a university hospital and from the local population through advertisements via social media (Instagram®, Facebook®), and local university radio.

### Randomization and blinding

Participants will be randomly allocated into three groups: the therapeutic pain neuroscience education (TPNE) group, the manual therapy (MT) group, and the multimodal (MM) group. Randomization will be performed by opening previously enumerated opaque envelopes. Each brown, opaque envelope will contain a paper identifying the group the participant is to be allocated to. This distribution will be made in a sequential order, randomized on Excel® by an external researcher. Randomization will be performed in blocks of 15 envelopes with five of each available group to match the groups’ sample. Two physiotherapists will be responsible for the evaluations. They will be blinded to the treatment allocation group. A physiotherapist will also be responsible for the group interventions and will be blinded to the results obtained in the evaluations.

### Data collection

Potentially eligible subjects, who have signed the consent form to participate in this trial, will fill out a form for collecting sociodemographic and anthropometric information, such as weight and height, and headache characteristics, such as pain intensity and disease time (in years). All patients will receive a headache diary, which should be filled out daily for 30 days, thus providing baseline data for the frequency and intensity of migraine attacks.

**Fig. 1:**
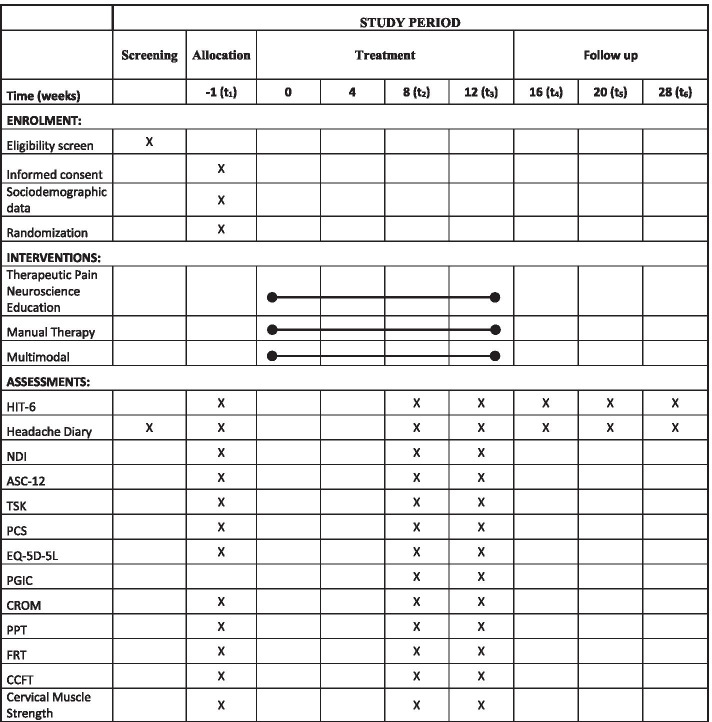
Study schedule summary. Legend: *HIT-6* Headache Impact Test; *NDI* Neck Disability Index; *ASC-12* 12 item Allodynia Symptom Checklist; *TSK* Tampa Scale for Kinesiophobia; *PCS* Pain Catastrophizing Scale; *EQ-5D-5L* 5-level EQ-5D tool for measuring the quality of life; *PGIC* Patient Global Impression of Change Scale; *CROM* Cervical Range of Motion; *PPT* Pressure Pain Threshold; *FRT* Flexion Rotation Test; *CCFT* Craniocervical Flexion Test.  Treatment time (12 weeks)

After 30 days, volunteers will return for the initial assessment, which includes questionnaires and physical examination, as described in Fig. 1. After the first assessment, the participants will be randomized. Throughout treatment, the headache diary must be filled out monthly to assess the frequency and intensity of attacks. Participants will be reevaluated at the end of the fourth, eighth, and twelfth weeks of treatment. These assessments will follow the same protocol as the initial assessment, including the Patient Global Impression of Change Scale [35]. All primary and secondary outcomes will be evaluated at the fourth, eighth, and twelfth weeks of the treatment period. Follow-up will then occur at 1, 2, and 4 months after the end of the treatment period to assess headache-related disability, in addition to frequency (days/month) and intensity of headache, using data from the headache diary.

### Questionnaires

- *Headache Impact Test* (HIT-6) is a tool assessing the headache impact with good psychometric properties among patients with headache [36]. The final score varies between 36 and 78 and is classified as little or no impact (≤ 49 points), some impact (50–55 points), considerable impact (56–59 points), and severe impact (≥ 60 points) [37]. For headache disability, good results will be attributed to those individuals with a reduction ≥ 6 points, which is the MCID for this scale [34].
- *Neck Disability Index* (NDI) is a questionnaire assessing neck pain-related limitations in daily living activities such as personal care, sleep, and reading. The score ranges from 0 to 50 points, and the individual can be classified as: without disability (0–4 points), mild disability (5–14 points), moderate disability (15–24 points), severe disability (25–35 points), and total disability (> 36points) [38, 39]. This tool is valid and reliable, with good responsiveness [40, 41].
- *12 item Allodynia Symptom Checklist* (ASC-12) [42] is a 12-item questionnaire to identify the presence of and classify the severity of cutaneous allodynia based on discomfort or pain felt during migraine crisis to perform activities of daily living, such as combing the hair or wearing glasses. The score can be up to 24 points and is classified as: no allodynia (0–2 points), mild allodynia (3–5 points), moderate allodynia (6–8 points), or severe allodynia (≥ 9 points). Florencio et al. (2012) translated and validated the Brazilian version of this tool, which is evidenced as quick, practical, and reliable for the evaluation of cutaneous allodynia [43].
- *The Tampa Scale for Kinesiophobia* (TSK) is a 17-item scale originally developed to measure the fear of movement, with a total score between 17 and 68 points [44]. This questionnaire has good reliability and psychometric applicability for the cervical region, and a correlation with depression and catastrophic symptoms [45].
- *Pain Catastrophizing Scale* (PCS) [46] is a 13-item questionnaire that measures pain-related catastrophizing and consists of three subscales: assessing helplessness, magnification, and rumination. The total score is calculated as the sum of all items, ranging from 0 to 52 points [47]. It has been demonstrated to be a reliable and valid measure of catastrophizing [46].
- EQ-5D-5L [48] is a version of the 5-level EQ-5D tool used to assess health-related quality of life [49]. This version comprises five dimensions: mobility, self-care, usual activities, pain/discomfort, and anxiety/depression. The version also includes the visual analog scale of the EQ (EQ-VAS), which records the patient’s self-assessment of health from “the best health you can imagine” to “the worst health you can imagine.”
- *The Patient Global Impression of Change Scale* (PGIC) is used to quantify individuals’ perception of worsening or improvement. The PGIC is a scale of seven items in which individuals rate their improvement in association with an intervention. The patients’ classification is present from 1 (no change or worsening) to 7 (a great deal better) [35].

### Pressure pain thresholds

The pressure pain threshold (PPT) will be assessed using a digital manual dynamometer (DDK-20 Kratos®) adapted for algometry, with participants in the sitting position. The PPT is a measure that has good reliability in healthy adults in the cervical region [50]. The trained examiner will apply a constant pressure of 1.0 kg/cm^2^/s with the device optimally positioned perpendicular to the anatomical surfaces to be evaluated. A digital metronome with a frequency of 1 Hz will be used in all evaluations to provide audio feedback and to standardize the speed of pressure application. Participants will be asked to signal as soon as they first feel pain. Pressure pain thresholds will be assessed randomly, and bilaterally over the upper trapezius, temporal, sternocleidomastoid, suboccipital, anterior scalene, and levator scapulae muscles, three times.

### Cervical range of motion

Cervical range of motion and cervical muscle strength will be evaluated using the Multi-Cervical Unit equipment (MCU, BTE Technologies, Inc., 2006). This tool has excellent reliability for all movements (ICC, 0.82 to 0.96) [51]. Two trials will be collected for each movement (flexion, extension, lateral flexion, and rotation) to determine the cervical range of motion with the volunteer in a sitting position. The head brace will be positioned comfortably around the volunteer’s head.

### Cervical muscle strength

To measure cervical muscle strength with the MCU, a load cell will be plugged into the brace that was used to measure the isometric force applied during cervical flexion, extension, and lateral flexion movements. There will be familiarization with the test by submaximal contraction of flexion and extension before the beginning of data collection. The evaluator will then request maximal isometric voluntary contractions through three repetitions of 3 s for each of the muscle groups. There will be a 20-s interval between each repetition and a 3-min interval between each movement to avoid muscle fatigue. The strength assessment of this equipment presents excellent reliability for all movements (ICC, 0.923–0.99) [51].

### Flexion rotation test (FRT)

Restricted movements of the upper cervical spine (C1-C2 segment) will be evaluated by the flexion rotation test (FRT) using the Cervical Range of Motion (CROM®) device over to the patient’s head. Participants will be positioned in the supine position with the head supported by the examiner’s hand, which will perform maximum passive flexion of the cervical spine followed by the maximum passive cervical rotation on each side [52–54]. The final range of motion is determined when the examiner perceives resistance or when the patient reports pain in the upper cervical segment. The FRT was considered positive when the subjects had a range of motion of less than 34° for right or left rotation [53, 54].

### Craniocervical flexion test (CCFT)

The craniocervical flexion test (CCFT) is a neuromuscular low-load test used to assess deep cervical flexor activation and endurance [55]. The test will be performed with the individual in a supine position, with a pressure biofeedback unit (Stabilizer Chattanooga South Pacific; USA) placed behind the neck and inflated up to 20 mmHg. Individuals will be instructed to perform a slow and gentle head-nodding action (craniocervical flexion) over five incremental stages (2 mmHg at each stage). Each stage will be maintained for 10 s. The participants will perform two 2-s repetitions at each stage to become familiar with the task. They will perform the CCFT by holding the target level for 10 s with a rest period of 30 s between levels without any compensation. The test will be interrupted when any compensation is identified, such as simultaneous palpable contraction of superficial flexors, and the latest stage performed without any compensation will be registered as the targeted stage [55].

### Statistical methods

A descriptive analysis of all variables will be performed, and the data will be reported as mean, standard deviation, and 95% confidence intervals. The statistical analysis will be performed according to the nature and distribution of each variable and be evaluated to verify any changes in the parameters and differences between the groups after the end of the protocol. For the analysis of outcomes, a significance level of 0.05 will be considered.

The intention-to-treat analysis will include all randomized volunteers, regardless of their treatment adherence in the evaluations and analyses [56]. The between-groups comparison will be performed using a mixed-effect regression model for all outcomes, considering the group effect, time effect, and group vs. time interaction. In addition, in those outcomes without predetermined MCID values, it will be calculated based on a distribution-based approach. The clinical relevance of the differences between groups will be classified according to the combination of MCID and effect size (ES), as suggested by Armijo-Olivo et al. [57]. The ES will be estimated by using Cohen’s d. The clinically relevant difference should present a mean difference that is more significant than MCID and ES > 0.4.

## Discussion

### Potential impact and significance of the study

The multimodal approach proposed in this clinical trial has been discussed to treat different dysfunctions of the neck [58], mainly because of its relationship with the cervical spine and the inclusion of education in patients with chronic disease [19]. However, there are no previous studies that used a multimodal physical therapy treatment for migraine, or evidence of this multimodal protocol’s effects compared to manual therapy techniques or therapeutic pain neuroscience education alone, separately. Due to the presence of cervical dysfunctions and musculoskeletal disorders [25, 59–61], and their psychosocial factors, there are reasons to infer that these individuals with migraine will benefit from this multimodal approach.

Regarding the treatments already described, Bevilaqua-Grossi et al. proposed adding a combination of manual therapy with trigger point release and cervical stretching to the pharmacological treatment, and observed clinical improvement in the frequency and intensity of migraine attacks when associated with the variables of global perception of change and satisfaction with treatment, as well as a significant increase in the pressure pain threshold when compared to the control group [9]. Despite the positive results, no other aspects of cervical dysfunctions that have already established in individuals with migraine, such as decreased cervical muscle strength [23], cervical disability [62–64], and the inclusion of therapeutic pain neuroscience education for the treatment of these patients were considered in this previous trial.

Migraine is accompanied by different musculoskeletal and functional dysfunctions of the cervical spine [25, 59–61], suggesting that patients will benefit from a multimodal treatment potentially including relaxation techniques, manual therapy that includes myofascial release, trigger point release, cervical mobilizations and stretching, and exercise programs of the cervical spine muscles, along with therapeutic pain neuroscience education in the same treatment protocol.

There is also little evidence regarding the self-management of migraines with home orientation [65]. We know that a program of therapeutic pain education on the disease, its pharmacological, nutritional, and exercise orientation, and stretching was effective in reducing the frequency of headache by 50% in 34% of patients, in addition to improvements in disability, quality of life, self-efficacy, and satisfaction with treatment [66]. Thus, a 50% reduction in the frequency of pain was observed when patients underwent self-administered message-based behavioral interventions [67]. However, little is currently known about the effectiveness of a self-approach to migraine management, including physiotherapeutic orientation, and how much this approach could contribute to patient improvement. If these approaches are more effective together than separately, then consequently, we hope to find a greater reduction in headache-related disability, one of the most addressed issues in the literature due to the high disability rate present in the migraine population [68]. The results will be informed to participants, healthcare professionals, the public, and other groups through articles published in journals of major impact, social media of our search group, lectures for the community, and national and international events.

### Limitations

It should be recognized that this protocol has some limitations. First, patients will be recruited from both tertiary headache centers and the general population. The sample may be composed of cases with distinct severities of migraine. Therefore, there must be a minimum of 3 days of headache per month, and those with concomitant headache diagnoses should be excluded. In addition, due to the greater female preponderance observed in migraine [68], we may have more women than men included in this study. As we will not control for gender distribution among the groups, we cannot guarantee the ability to demonstrate gender differences in the response to treatment. Moreover, the allocation sequence generation and concealment used should guarantee an appropriate randomization process, which avoids the influence of either known or unknown prognostic factors [69].

Second, it will not be possible to blind the participants and the therapist who will apply the interventions, considering the proposed treatment approach. This would increase the risk of bias due to deviation from the intended intervention, affecting mainly the participant’s adherence [69]. According to the Cochrane risk of bias assessment [69], if participants are aware of their assigned intervention, it is more likely that health-related behaviors will differ between the intervention groups. However, the planned interventions of this protocol itself expect different behaviors from the participants (active or passive behaviors). The co-intervention using the prescribed medication is also expected and controlled in the current study by the use of a headache diary. Patients who start a new medical treatment during the trial screening and selection will be recruited to start the study protocol only after 3 months, which is the time required to stabilize the medication’s effects. The patients will be instructed to use only the prescribed medications when appropriate, but the use of non-prescribed medications should also be mentioned separately in the pain diary.

Finally, the results of this trial will be restricted to the immediate effects on most of our variables and mid-term effects on clinical outcomes (headache impact, frequency, and intensity). This was a methodological choice to avoid a higher proportion of dropouts in this trial. However, we prioritized patient-reported outcomes related to migraine.

### Contributions to physical therapy

The results of this clinical trial will help elucidate the effects of a multimodal protocol and highlight if it has advantages in relation to the isolated effect of passive and active strategies in patients with migraine. The physical therapy approach for patients with migraine has been classically and mainly composed of manual therapy. Still, the literature regarding wholly passive treatments is controversial, despite good patient perceptions of improvement. In addition, much has been said about biopsychosocial approaches for patients with chronic pain, including migraine.

However, there is still no description in the literature of a treatment approach that includes physiological and musculoskeletal factors along with patient education of the disease and self-management of migraine. The complexity of migraine in terms of clinical presentation and the influence of physical and psychological comorbid conditions on its prognosis [1, 2] indicates that therapeutic approaches should be multidimensional.

Moreover, cervical dysfunction in migraine patients is characterized by signs and symptoms such as neck pain, decreased muscle strength, altered motor control, hypersensitivity, and reduced range of motion [59–64]. We hypothesized that including therapeutic modalities that not only facilitate analgesic effects, but also aim to enhance muscle capacity and promote more accurate self-management of the disease, would provide prolonged effects in the management of migraine.

### Trial status

The protocol was started in August 2020, and approval from the ethics committee was obtained in September 2020. Patient recruitment will begin in April 2021 and is expected to be completed in January 2023. The study will be completed in August 2023.

## Data Availability

Data sharing is not applicable to this article as no datasets were generated or analysed during the current study.

## Abbreviations

ASC-12: 12-Item Allodynia Symptom Checklist
CCFT: Craniocervical Flexion Test
CROM: Cervical Range of Motion
EQ-5D-5L: 5-Level EQ-5D tool for measuring the quality of life
ES: Effect Size
FRT: Flexion Rotation Test
IHS: International Headache Society
HIT-6: Headache Impact Test
HCFMRP-USP: *Hospital das Clínicas* Of the *Ribeirão Preto* Medical School of the University of São Paulo
MCID: Minimum clinically important difference
MCU: Multi-Cervical Unit
MM: Multimodal
MT: Manual therapy
NDI: Neck Disability Index
PCS: Pain Catastrophizing Scale
PGIC: Patient Global Impression of Change Scale
PPT: Pressure Pain Threshold
REBEC: *Registro Brasileiro de Ensaios Clínicos*
TPNE: Therapeutic Pain Neuroscience Education
TSK: Tampa Scale for Kinesiophobia
